# Hapten-Specific Cellular Immune Responses in the Elicitation and Sensitization Phases of Murine Contact Hypersensitivity

**DOI:** 10.3390/biom15111540

**Published:** 2025-11-01

**Authors:** Kornél Molnár, Gábor Kovács, Bence Kormos, Petra Aradi, Zoltán Jakus

**Affiliations:** Department of Physiology, Semmelweis University, H-1094 Budapest, Hungary; molnar.kornel@semmelweis.hu (K.M.); kovacs.gabor@semmelweis.hu (G.K.); kormos.bence04@stud.semmelweis.hu (B.K.); aradi.petra@semmelweis.hu (P.A.)

**Keywords:** contact hypersensitivity, mouse models, inflammation, haptens, TNCB, DNFB, dermatitis

## Abstract

Contact dermatitis (CD) is a common inflammatory skin condition with irritant etiology or a delayed-type hypersensitivity called allergic contact dermatitis (ACD). Contact hypersensitivity (CHS) is a widely used rodent model of ACD and similarly consists of two phases: sensitization and elicitation. To trigger CHS, low-molecular-weight haptens, such as DNFB or TNCB, are commonly applied. However, the characterization of the induced immune response remains incomplete. Our aim was to characterize the immune response after first and repeated exposures to model haptens. First exposure to DNFB or TNCB led to significant ear swelling, with DNFB causing a more pronounced effect. DNFB enhanced neutrophil infiltration, whereas TNCB led to macrophage, dendritic cell, and helper T cell accumulation. Repeated DNFB exposure did not aggravate edema significantly, while TNCB re-exposure enhanced edema formation and induced neutrophil granulocyte, dendritic cell, and helper and cytotoxic T cell accumulation. Our results demonstrate that a hapten-specific immune response is induced during both phases of CHS. A detailed understanding of allergen-specific immune responses is crucial for the appropriate selection of the model and for gaining deeper insight into the mechanisms of inflammatory skin diseases. These findings may contribute to the targeted selection of haptens.

## 1. Introduction

Contact dermatitis (CD) is a common inflammatory skin disease that affects 5–20% of the global population. Clinically, CD is characterized by erythema, the formation of papules and vesicles, along with pruritus, rash, and itchy skin. This condition can develop due to different etiologies, most commonly irritant contact dermatitis (ICD)—approximately 80% of the cases—or allergic contact dermatitis (ACD), among other subtypes [1].

ICD is a nonspecific inflammatory reaction of the skin to chemical irritants. The development depends on multiple environmental and endogenous factors, as well as on the irritant potential of the agent. The pathomechanism of ICD involves the penetration of the skin by the irritant, inducing damage to the local microenvironment, which triggers keratinocytes and other resident cells to secrete pro-inflammatory cytokines, resulting mainly in the infiltration of the cells of innate immunity [1,2,3].

In contrast to ICD, allergic contact dermatitis is a delayed-type (or type IV hypersensitivity) reaction that develops upon repeated exposure to specific haptens. In humans, these may include various metals (e.g., chromium, nickel, iron, cobalt), preservatives found in certain cosmetics (e.g., p-phenylenediamine, benzophenones), as well as drugs such as penicillin or benzocaine [2,4,5]. These agents are low-molecular-weight haptens (<1000 Da) that are not immunogenic by themselves but elicit adaptive immune responses when conjugated to larger carrier proteins in the skin [6,7]. ACD has two immunologically distinct phases. During the sensitization phase, the skin is exposed to the hapten for the first time. Following the hapten–carrier complex formation, professional antigen-presenting cells—including Langerhans cells (LCs) and dermal dendritic cells—take up and process these and then proceed to initiate the activation and maturing of hapten-specific T cells [8]. Re-exposure to the same hapten induces the elicitation phase, which leads to the recruitment of effector T cells and cells of innate immunity, resulting in a prolonged inflammatory response [7,9]. Classically, this T cell-mediated response is categorized as delayed-type (type IV) hypersensitivity; however, a more recent paradigm spearheaded by Werner Pichler further classified it based on the characteristic cytokine and chemokine secretion patterns and recruited immune cells. These subdivisions include type IVa reactions with macrophages and monocytes being preferentially activated, type IVb with Th2 dominance and eosinophil activation, and type IVd with increased neutrophil involvement. The type IVc subcategory is characterized by cytotoxic T cell activity, which is usually present in all type IV reactions [10,11]. Notably, the description of these responses was based mostly on human data, and little is known about their presence in animal models of contact dermatitis.

Contact hypersensitivity (CHS) represents a well-established model of ACD that can be induced in various animal species, including mice and rats, through the topical application of contact sensitizers to the skin [12,13,14,15,16]. This model is not only useful for studying ACD but also serves as a valuable tool for investigating the distinct phases of immunization and the effector response. Several chemical compounds are commonly used to induce CHS in experimental models, including 1-fluoro-2,4-dinitrobenzene (DNFB), 2-chloro-1,3,5-trinitrobenzene (TNCB), fluorescein isothiocyanate (FITC), and oxazolone [7]. These haptens are often used as models for ACD, and some elicit strong ICD-like response upon first contact; however, these could differ. For example, Bennett and colleagues reported that, in a transgenic mouse model, the ablation of LCs resulted in a diminished CHS reaction using TNCB or oxazolone, while Kissenpfennig et al. employed a similar transgenic mouse strain but found no effect on the severity of inflammation after repeated DNFB application, highlighting the potential differences in the immunization to these haptens [16,17,18]. Sporadic, more-recent studies suggest hapten-dependent differences, in which TNCB-induced CHS is dependent on tumor necrosis factor receptor 1 (TNFR1) signaling, while DNFB exhibits a paradoxical TNFR1 effect in which TNFR1 deficiency reduces necrosis but increases edema, indicating distinct tissue damage mechanisms [19]. Furthermore, it has been demonstrated that the intensity of inflammation elicited upon the first contact with DNFB correlates with the severity of CHS after repeated exposure, suggesting a dose-dependent difference in the local immune processes [20].

In addition, it was reported almost four decades ago that performing a challenge with TNCB on the ear of DNFB-sensitized rats results in a more prominent swelling than that on hapten-naïve animals suggesting some form of cross-reaction [21]. Meanwhile, in a recent study by our research group, we found that a DNFB challenge in TNCB-sensitized mice did not result in a similar response, implying that these cross-reactions are not universal [22]. Collectively, more-detailed studies are needed to characterize the effects of TNCB and DNFB on the immune response in CHS.

Here, we aimed to characterize the local processes partaking in the inflammatory response during first and repeated exposures to TNCB and DNFB. First, we examined the irritant effects of these haptens by painting the ears of wild-type C57BL/6 mice with TNCB or DNFB solution and then assessed the inflammatory responses 24 h post-exposure. Following that, we characterized the hapten-specific immune responses in the CHS model. Our results show that TNCB and DNFB elicit markedly different inflammatory processes both after first as well as after repeated exposures. In our experiments, DNFB induced significant irritant responses while the delayed-type hypersensitivity reaction was less prominent after repeated exposure. On the other hand, first exposure to TNCB resulted in a mild irritant response but elicited a strong, hapten-specific response in the effector phase. Our results highlight that these haptens have significant differences in the elicited immune response, and thus, the selection of the appropriate allergen is a critical factor that can influence the outcomes of studies employing the CHS model.

## 2. Materials and Methods

### 2.1. Experimental Animals

In our experiments, 8–12-week-old C57BL/6 wild-type mice were used. Each experimental group included both male and female animals with a close-to-equal ratio. The total number of animals in all the experiments presented is 98. Inclusion criteria were determined based on animal age and sex; no animal or data point was excluded. All animal experiments were approved by the Animal Experimentation Review Board of the Semmelweis University and the Government Office for Pest County (PE/EA/00658-6/2023 and PE/EA/00659-6/2023 both approved on 7 August 2023). Experimental animals were housed in either specific pathogen-free or conventional animal facilities with ambient temperature between 18 and 22 °C, 45% humidity, and 12/12 h dark–light cycles. Food and water were supplied ad libitum.

### 2.2. Single Antigen Exposure

Before the application of haptens, the initial ear thickness was measured using a caliper (Kafer Messuhrenfabrik GmbH -Villingen-Schwenningen, Germany). One ear of each mouse was painted with either acetone or a mixture of acetone with corn oil (3:1) as a vehicle control. The other ear was painted with either 20 µL of 3% TNCB (Sigma-Aldrich—St. Louis, MO, USA, 79874) diluted in acetone or with 20 µL of 0.5% DNFB (Sigma-Aldrich, D1529) diluted in a 3:1 acetone/corn oil mixture. Twenty-four hours after the challenge, the ear thickness was measured again. The increase in ear thickness was calculated as the difference between the measurements before and 24 h after the challenge. Examiners were aware of the treatment during ear measurements due to TNCB and DNFB both discoloring the skin of the animals. Alternatively, one hind paw of each mouse was painted with either 100 µL acetone or a mixture of acetone with corn oil (3:1) as a vehicle control. The other hind paw was painted with either 100 µL of 3% TNCB diluted in acetone or with 100 µL of 0.5% DNFB diluted in a 3:1 acetone/corn oil mixture. Twenty-four hours after the challenge, the popliteal lymph nodes were removed for further processing. Isoflurane (Baxter—Deerfield, IL, USA) was used to anaesthetize the mice during the procedure [22].

### 2.3. Repeated Antigen Exposure and CHS Model

First, the abdominal skin of mice was painted with 100 µL of 3% TNCB diluted in acetone, with 100 µL of 0.5% DNFB diluted in a 3:1 mixture of acetone and corn oil, with 100 µL of acetone, or with the 3:1 mixture of acetone and corn oil. Five days after the first exposure, the initial ear thickness was measured using a caliper. After that, 20 µL of 1% TNCB diluted in acetone or 20 µL of 0.3% DNFB diluted in a 3:1 acetone/corn oil mixture was epicutaneously applied on both ears of the mice as repeated exposure. Ear swelling was determined as described earlier. Isoflurane (Baxter) was used to anaesthetize the mice during the process. This CHS protocol is based on the previously described protocols [22,23].

### 2.4. Histological Procedures and Immunofluorescence Staining

Isolated tissues (ear, popliteal lymph nodes) were fixed in 4% paraformaldehyde (Sigma-Aldrich) overnight at 4 °C and then dehydrated in a series of ethanol solutions (50%, 70%, 95%, and 100%). Ear samples were placed in xylene twice for 20 min, while the lymph node samples were put in xylene twice for 10 min. All samples were embedded into paraffin (Leica—Wetzlar, Germany, 39601006) using a Leica EG1150H (Wetzlar, Germany) embedding station. Sections of 6–7 µm thickness were prepared using a Thermo Scientific microtome (HM340E—Waltham, MA, USA) and processed for Hematoxylin and Eosin (H&E) staining (Leica, 3801602E; Leica, 3801582E) or for immunofluorescence staining.

Immunofluorescence staining was performed using the following antibodies in 1:100 dilution, incubated overnight at 4 °C: anti-LYVE1 (R&D Systems—Minneapolis, MN, USA, AF2125), anti-Ly6G (BD Biosciences—Franklin Lakes, NJ, USA, 551459), anti-CD45 (Merck, Darmstadt, Germany, 05-1416). The applied secondary antibodies were diluted at 1:250, and the samples were incubated at room temperature for 1 h with the following: Alexa Fluor 488 goat anti-rat IgG (Invitrogen—Waltham, MA, USA, A11006), Alexa Fluor 488 donkey anti-goat IgG (Invitrogen, A11055), Alexa Fluor 568 donkey anti-goat IgG (Invitrogen, A11057). For mounting and nuclear staining, DAPI-containing mounting medium (Vector Laboratories—New Arc, CA, USA, H-1200-10) was used [24,25]. All applied antibodies were previously validated by comparing them with control stainings.

Microscopic images were captured using a Nikon ECLIPSE Ni-U microscope (Nikon—Tokyo, Japan) connected to a Nikon DS-Ri2 camera. The lymphatic vessel number, perimeter, and area (mean of all visible lymphatic vessels in one mouse ear section) were quantified using the NIS-Elements Imaging Software (5.02.00 (Build 1266)) (Nikon—Tokyo, Japan) from anti-LYVE1 fluorescent images taken with a 20× objective. Alternatively, anti-LYVE1 immunofluorescence sections were scanned with a PANNORAMIC^®^ MIDI III scanner using PANNORAMIC^®^ Scanner Software 5.0.0. (3DHISTECH—Budapest, Hungary), and lymphatic vessel parameters were quantified in SlideViewer 2.9.0 (3DHISTECH). Lymph node perimeter and area were quantified using the NIS-Elements Imaging Software (Nikon) from H&E-stained images taken with a 4× objective. The epidermis and dermis (mean of 10 individual measurements from one sample) were quantified in SlideViewer 2.9.0 (3DHISTECH) from H&E-stained sections scanned with a PANNORAMIC^®^ MIDI III scanner using PANNORAMIC^®^ Scanner Software 5.0.0. (3DHISTECH). Investigators performing the manual quantification were blinded to the treatment regimen.

### 2.5. Digestion of Histological Samples and Flow Cytometry

Ear skin or popliteal lymph node samples were collected, and the ear skin was cut into small pieces. The samples were then digested in 0.5 mL of digestion solution (0.4 mg/mL Collagenase D [Roche—Basel, Switzerland, 1108888201] and 5 mg/mL DNase I [Roche, 11284932001] dissolved in RPMI (containing glutamine, 10% fetal bovine serum, penicillin, streptavidin)) using a microtube Thermo-Shaker (BioSan TS-100, Riga, Latvia) for 30 min at 37 °C and 250 rpm. A single-cell suspension from the ear samples was prepared by passing the digested tissue through a 70 µm cell strainer (Falcon—Corning, NY, USA), while the digested lymph nodes were pressed through a 70 µm cell strainer using the rigid end of a syringe plunger. Samples were centrifuged for 10 min at 5000 rpm, and after discarding the supernatant, pellets were resuspended in a staining buffer (93% (*v*/*v*) PBS, 2% (*v*/*v*) heparin (TEVA Heparibene—Tel Aviv, Israel), and 5% (*v*/*v*) fetal bovine serum).

The single-cell suspensions were then stained with the following antibodies in a 1:200 dilution with the staining buffer, which also contained True-Stain Monocyte Blocker (BioLegend—San Diego, CA, USA, 426103) in a dilution of 1:20 and anti-CD16/32 (Invitrogen, 16-0161-86) in a dilution of 1:200: anti-CD45 PE (BD Bioscience—Franklin Lakes, NJ, USA, 553081), anti-Ly6G PerCP-Cy5.5 (BD Bioscience, 560602), anti-CD11b eFluor™ 450 (ThermoFisher—Waltham, MA, USA, 48-0112-82), anti-F4/80 APC-Fire750 (BioLegend, 123152), anti-CD11c APC (ThermoFisher, 17-0114 82). Alternatively, single-cell suspensions were stained with anti-CD45-PE (BD Bioscience, 553081), anti-CD3 PE-Cyanide 7 (BioLegend, 100220), anti-CD4 Pacific Orange (ThermoFisher, MCD0430), anti-CD8a Pacific Blue (ThermoFisher, MCD0828), anti CD25 APC (BioLegend, 406509), anti-CD19 FITC (BioLegend, 152404), and anti-CD69 PerCP-Cy5.5 (BD Bioscience, 55113), in a 1:200 dilution in the presence of True-Stain Monocyte Blocker (1:20) and anti-CD16/32 (1:200). The samples were incubated in the antibody-containing solution for 1 h at 4 °C. Following a washing step, pellets were resuspended in 0.5 mL PBS and analyzed using a Beckman Coulter CytoFLEX S cytometer (Brea, CA, USA) for 5 min at a flow rate of 30 µL/min. The applied gating strategy is available as part of the Supplementary Materials (Supplementary Figure S1).

### 2.6. Presentation of Data and Statistical Analysis

Microscopic image processing and analysis were performed using Nikon NIS-Elements Imaging Software (BR4.60.00) or alternatively using PANNORAMIC^®^ Scanner Software 5.0.0. (3DHISTECH) and SlideViewer 2.9.0 (3DHISTECH), Adobe Illustrator (29.8.1), and Adobe Photoshop (CS6). Flow cytometry data were evaluated using CytExpert software (2.3) (Beckman Coulter—Brea, CA, USA). Experiments were performed for the number of times indicated in the figure legends. Scatter plots show the means and SEMs of all mice or samples from the indicated number of independent experiments. The effect of the first and second antigen exposure on ear thickness or immune cell infiltration was calculated by subtracting the mean of the corresponding control group from each individual data point after repeated exposure. Statistical analysis was performed in GraphPad Prism 7 and Microsoft Excel (Office 365). Normal distribution of all datasets was assessed using the Shapiro–Wilk test. Differences between groups were evaluated using an unpaired Student’s *t*-test or two-way ANOVA followed by Tukey’s multiple comparisons post hoc analysis. Datasets not following normal distribution were analyzed with Mann–Whitney U test for two-group comparisons and Kruskal–Wallis test for multiple groups. An α < 0.05 was considered statistically significant.

## 3. Results

### 3.1. A Single Exposure to DNFB Induces a More Pronounced Local Inflammatory Response than a Single TNCB Application

**Figure 1:**
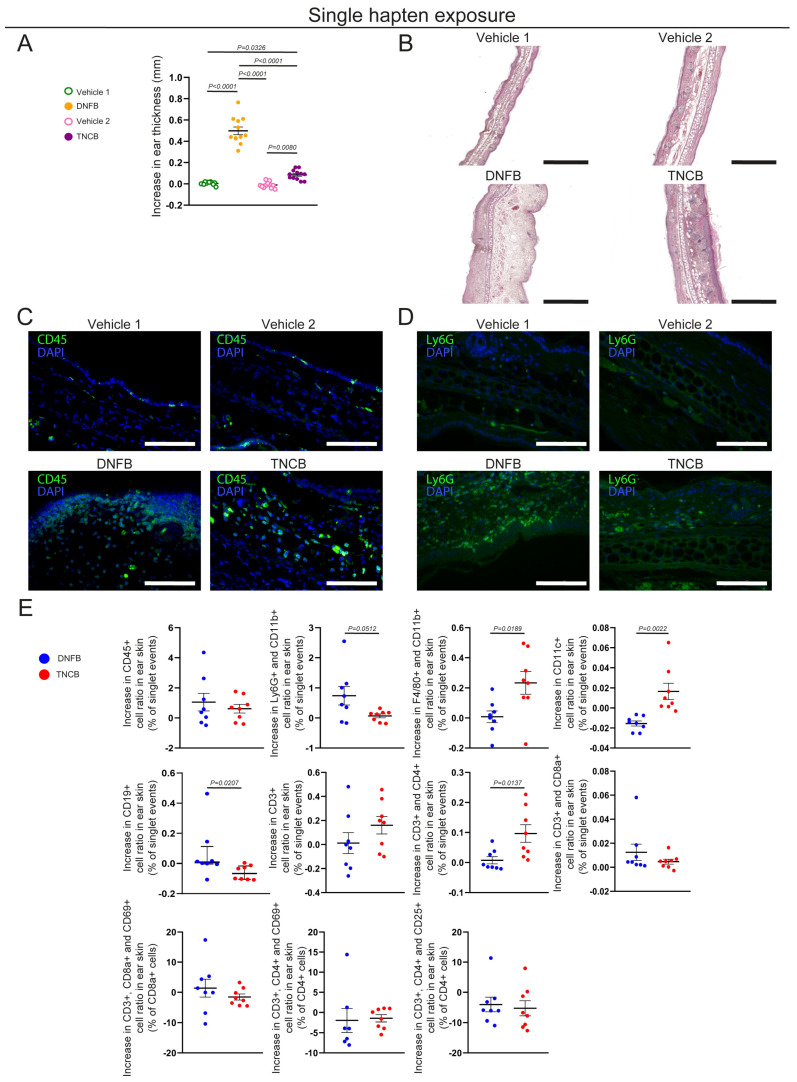
Characterization of the immune response in the ear skin after a single exposure to TNCB or DNFB. Young adult C57BL/6 mice were painted with 3% TNCB or 0.5% DNFB solutions or with vehicle on the ear. Ear thickness was measured before and 24 h after the application. (**A**) The increase in ear thickness of mice 24 h after a single hapten exposure (two-way ANOVA; mean ± SEM; *n* = 12 animals for each group). (**B**) H&E staining of paraffin-based sections of ears 24 h after a single exposure (bars = 500 µm; *n* = 4 animals for each group). (**C**) Anti-CD45 immunofluorescence staining of paraffin-based sections of ears 24 h after a single exposure (bars = 100 µm; *n* = 4 animals for each group). (**D**) Anti-Ly6G immunofluorescence staining of paraffin-based sections of ears 24 h after a single exposure (bars = 100 µm; *n* = 4 animals for each group). (**E**) Quantitative data from flow cytometry analysis of digested ear samples 24 h after a single exposure. Data was normalized to controls by subtracting the mean value of the corresponding control group from each individual data point (unpaired *t*-test; mean ± SEM, in case of “Increase in CD19+ cell ratio in ear skin (% of singlet events)”: Mann–Whitney test, Median ± IQR; *n* = 8 animals for each group).

First, we aimed to characterize the local immune processes elicited by a single exposure to TNCB and DNFB. To this end, we topically painted the ears of C57BL/6 mice with acetone-based hapten solutions. Ear thickness was measured before and 24 h after the application, and we found that both TNCB and DNFB induced significant local swelling compared with controls. Additionally, a single DNFB exposure induced significantly greater local swelling than TNCB (Figure 1A). Examination of H&E-stained ear sections showed substantial cell infiltration in both immunized groups compared with the corresponding controls. The epidermal layer was significantly thicker in TNCB-exposed ears, while the dermis was significantly larger in the DNFB-exposed ones (Figure 1B and Supplementary Figure S2A). We detected substantial CD45+ immune cell infiltration in both hapten-exposed groups, while Ly6G+ neutrophil granulocyte infiltration was more prominent in DNFB-exposed ears with immunofluorescence staining (Figure 1C,D).

Differences in the infiltrating immune cell populations were quantified with flow cytometry, and groups with single TNCB or DNFB exposure were normalized to their corresponding control groups. No significant difference was observed in total CD45+ leukocyte ratios between the TNCB- and DNFB-exposed ears; however, a tendency towards greater Ly6G+, CD11b+ neutrophil infiltration was detected in the DNFB-exposed group than that in the TNCB-painted ones. In addition, a significantly higher ratio of F4/80+, CD11b+ macrophages, and CD11c+ dendritic cells was observed in TNCB-immunized ears. The CD19+ B cell population was more numerous in the DNFB-exposed ears. No significant difference was found in total CD3+ T cell ratio, but CD3+, CD4+ helper T cell infiltration was significantly elevated in response to a single TNCB exposure. No significant difference was found in CD3+ CD8a+ cytotoxic T cell, CD4+ CD25+ regulatory T cell, or activated helper (CD3+, CD4+, CD69+) or cytotoxic (CD3+, CD8a+, CD69+) T cell ratios (Figure 1E).

Additionally, dermal lymphatic vessel morphology was assessed using anti-LYVE1 immunofluorescence images of the ears after a single exposure to TNCB or DNFB. No significant difference was found in the number of lymphatic vessels between these groups; however, the mean area of lymph vessels was significantly larger in the DNFB-painted ears than in TNCB-exposed ones (Supplementary Figure S2B,C).

**Figure 2:**
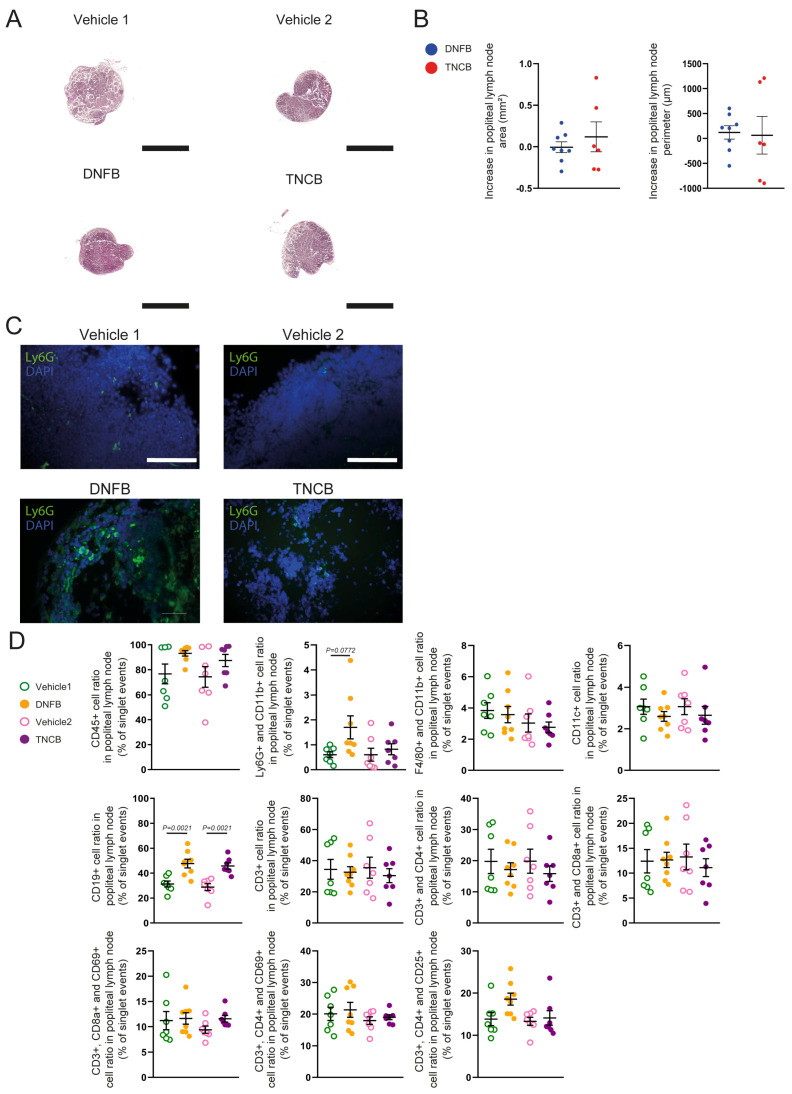
Characterization of the draining popliteal lymph node after a single TNCB or DNFB exposure. Young adult C57BL/6 mice were painted with 3% TNCB or 0.5% DNFB solutions or by the vehicles on the hind paw and popliteal lymph nodes were collected 24 h later. (**A**) H&E staining of paraffin-based sections of popliteal lymph nodes 24 h after single exposure (bars = 1 mm; *n* = 6 lymph nodes for the TNCB and vehicle 1 groups, *n* = 7 for the vehicle 2 group, and *n* = 8 for the DNFB group). (**B**) Quantification of the popliteal lymph node area and perimeter 24 h after single exposure, based on H&E-stained slides. Data was normalized to controls by subtracting the mean value of the corresponding control group from each individual data point (unpaired *t*-test; mean ± SEM; *n* = 6 lymph nodes for the TNCB group and *n* = 8 for the DNFB group). (**C**) Anti-Ly6G immunofluorescence staining of paraffin-based sections of popliteal lymph nodes 24 h after single exposure (bars = 100 µm; *n* = 6 lymph nodes for the TNCB and vehicle 1 groups, *n* = 7 for the vehicle 2 group, and *n* = 8 for the DNFB group). (**D**) Quantitative data from flow cytometry analysis of digested popliteal lymph node samples collected 24 h after a single exposure (two-way ANOVA; mean ± SEM; *n* = 7 lymph nodes for the vehicle 1, vehicle 2, and TNCB groups and *n* = 8 for the DNFB group).

Following that, we aimed to characterize the changes in regional lymph node morphology and cellular composition in response to a single TNCB or DNFB exposure. To accomplish this, C57BL/6 mice were painted with TNCB or DNFB solution or with their vehicle controls on the hind paw, and the popliteal lymph nodes were harvested 24 h after the exposure. Paraffin-based sections of the lymph nodes were examined with H&E staining, showing no clear histomorphological differences (Figure 2A).

The area and perimeter of the lymph node sections were quantified, and no differences were detected (Figure 2B). Examining the slides with immunofluorescence staining uncovered that Ly6G+ neutrophils infiltrated the popliteal lymph nodes after both TNCB and DNFB exposure, whereas these cells were not visualized in the control lymph nodes (Figure 2C). Upon quantification of the cellular components of the lymph node with flow cytometry, no differences were observed in the total CD45+ leukocyte population between TNCB- and DNFB-exposed groups. However, DNFB application showed a tendency towards an increased Ly6G+, CD11b+ neutrophil ratio compared with that in the vehicle controls, and both haptens caused a significant increase in the CD19+ B cell ratio compared with their respective controls. No differences were observed in other immune cell populations (Figure 2D).

### 3.2. DNFB Exposure Induces Edema Formation While Repeated TNCB Application Elicits Local Leukocyte Accumulation

**Figure 3:**
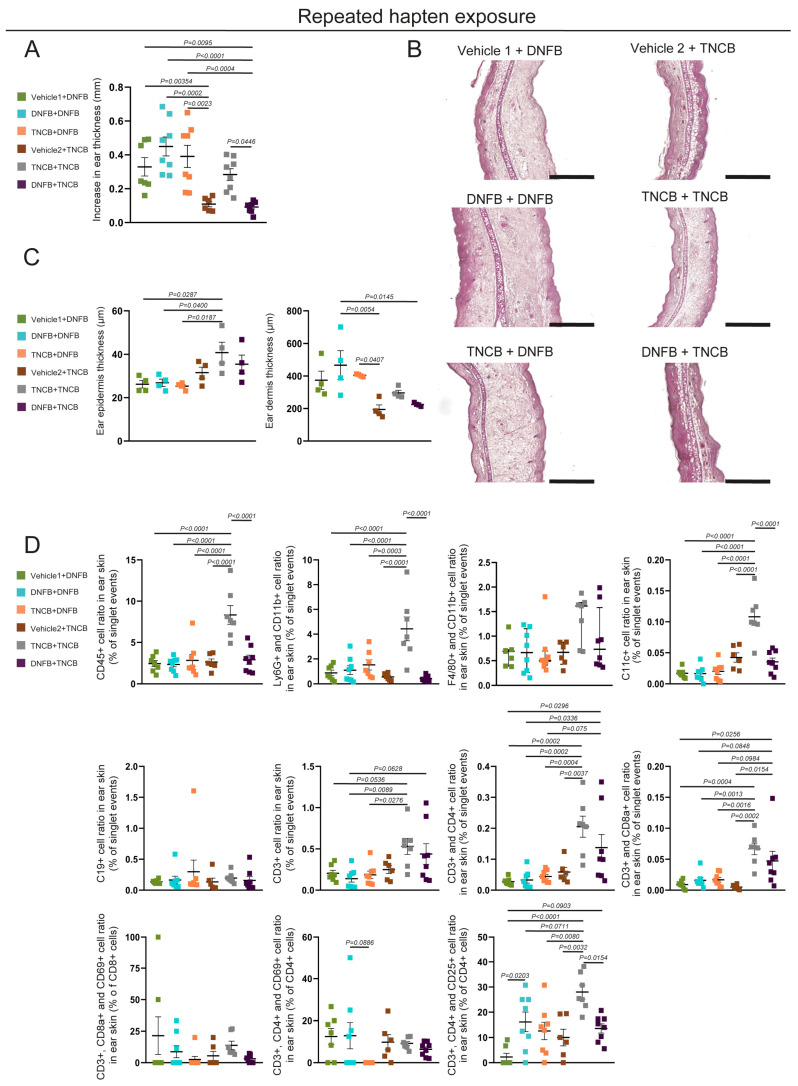
Characterization of the hapten-dependent contact hypersensitivity reaction 24 h after challenge. The abdominal skin of young adult C57BL/6 mice was painted with 3% TNCB or 0.5% DNFB solutions or with vehicles. Five days after that, the ear thickness was measured, and the ears were painted with 1% TNCB or 0.3% DNFB solution. The reaction was assessed 24 h after the challenge. (**A**) The increase in ear thickness of mice 24 h after hapten challenge (two-way ANOVA; mean ± SEM; *n* = 6 animals for the vehicle 2 + TNCB group; *n* = 7 for the vehicle 1 + DNFB group; and *n* = 8 for the DNFB + DNFB, TNCB + DNFB, TNCB + TNCB, and DNFB + TNCB groups). (**B**) H&E staining of paraffin-based sections of ears 24 h after hapten challenge (bars = 500 µm; *n* = 6 animals for the vehicle 2 + TNCB group; *n* = 7 for the vehicle 1 + DNFB and TNCB + TNCB groups; and *n* = 8 for the DNFB + DNFB, TNCB + DNFB, and DNFB + TNCB groups). (**C**) Quantification of the dermis and epidermis layers from H&E-stained sections of mouse ears 24 h after the challenge (two-way ANOVA; mean ± SEM; *n* = 4 animals for each group). (**D**) Quantitative data from flow cytometry analysis of digested ear samples collected 24 h after the challenge (two-way ANOVA; mean ± SEM, in case of “Increase in F4/80+ and CD11b+ cell ratio in ear skin (% singlet events)”: Kruskal–Wallis test, Median ± IQR; *n* = 6 animals for the vehicle 2 + TNCB group; *n* = 7 for the vehicle 1 + DNFB and TNCB + TNCB groups; and *n* = 8 for the DNFB + DNFB, TNCB + DNFB, and DNFB + TNCB groups).

Next, we characterized the local immune response to repeated hapten exposure and the specificity of the inflammatory processes. To this end, we painted the shaved abdominal skin of C57BL/6 mice with TNCB or DNFB solutions or with vehicles as controls. Five days following that, we applied either TNCB or DNFB to the ears and examined the response 24 h later. Upon measuring the ear thickness, we found that the DNFB challenge induced a similar increase regardless of the sensitizing hapten. In contrast, a major increase after TNCB challenge was observed only in animals previously sensitized with TNCB (Figure 3A). The examination of the H&E-stained sections of the ears revealed that the epidermal layer thickened significantly upon TNCB re-exposure compared with that on DNFB application. Additionally, we observed a significantly thickened dermal layer after topical DNFB application with either DNFB or TNCB sensitization compared with local TNCB application in hapten-naïve animals (Figure 3B,C).

Quantification of infiltrating immune cells with flow cytometry revealed that repeated exposure to DNFB did not result in increased CD45+ immune cell infiltration compared with mice with first exposure or with previous TNCB sensitization. In contrast, repeated TNCB exposure significantly increased the local CD45+ leukocyte ratios compared with both vehicle-painted and DNFB-sensitized, then TNCB-exposed mice. Similarly, repeated contact with TNCB significantly elevated the local Ly6G+, CD11b+ neutrophil, and CD11c+ dendritic cell ratios compared with those in all other groups, whereas repeated DNFB exposure did not affect these populations (Figure 3D). Similar differences in the number of CD45+ cells and Ly6G+ neutrophils were observed in immunofluorescence staining of ear sections (Supplementary Figure S3A,B). Furthermore, no significant differences were found in the F4/80+, CD11b+ macrophage ratios. However, the CD3+ T cell; the CD3+, CD4+ T helper; and the CD3+, CD8a+ cytotoxic T cell ratios also significantly increased after repeated TNCB application, compared with re-exposure to DNFB. Interestingly, CD3+ T cells showed an increasing tendency when DNFB-sensitized mice were exposed to TNCB, compared with repeated DNFB application. In addition, TNCB exposure after DNFB sensitization caused a significant increase in CD3+, CD4+ T helper cell ratios compared with both first and repeated DNFB exposures. The CD3+, CD8a+ cell ratios were significantly increased after TNCB exposure in previously DNFB-sensitized mice compared with hapten-naïve mice exposed to either TNCB or DNFB and showed an increasing tendency relative to repeated DNFB application or DNFB exposure after TNCB sensitization. The CD3+, CD4+, CD25+ regulatory T cell ratios were significantly elevated after repeated DNFB or TNCB exposure, compared with those in groups with a single exposure to these haptens, while repeated TNCB application showed an increasing tendency compared with repeated DNFB exposure (Figure 3D).

**Figure 4:**
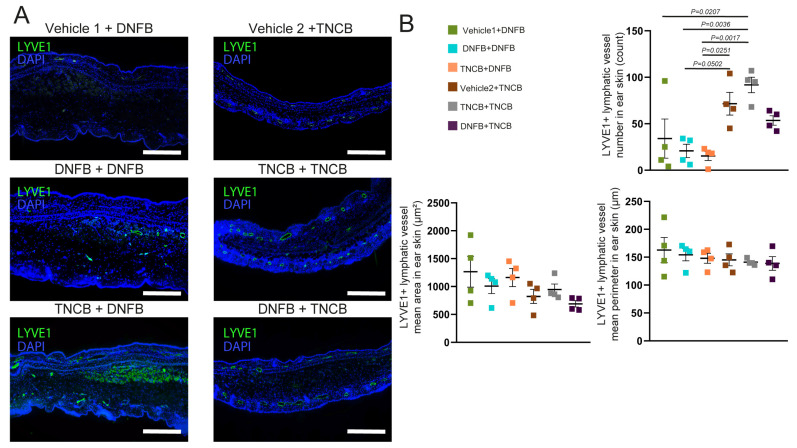
Characterization of dermal lymphatic vessels in the hapten-dependent contact hypersensitivity reaction 24 h after challenge. The abdominal skin of young adult C57BL/6 mice was painted with 3% TNCB or 0.5% DNFB solutions or with vehicles. Five days after that, the ear thickness was measured, and the ears were painted with 1% TNCB or 0.3% DNFB solution. The reaction was assessed 24 h after the challenge. (**A**) Anti-LYVE1 immunofluorescence staining of paraffin-based sections of ears 24 h after the challenge (bars = 100 µm; *n* = 4 ears for each group). (**B**) Quantification of lymphatic vessel mean perimeter, mean area, and number from anti-LYVE1 immunofluorescence images of ears collected 24 h after the challenge (two-way ANOVA; mean ± SEM; *n* = 4 ears for each group).

The changes in local lymphatic vessels were also investigated. We found that repeated TNCB exposure significantly increased lymphatic vessel numbers compared with repeated DNFB application. No significant differences were observed in lymphatic vessel perimeter or area (Figure 4A,B).

## 4. Discussion

Here we studied the local histological changes and infiltrating immune cells after a single and repeated exposures to TNCB and DNFB, two haptens commonly used in CHS rodent models of ACD. In our experiments, a single DNFB exposure elicited immense edema formation, mostly affecting the dermis, which was accompanied by neutrophil infiltration. In contrast, a single TNCB exposure also induced swelling, though significantly less than that for DNFB, and was associated mostly macrophage, dendritic cell, and CD4+ helper T cell infiltration (Figure 1A–E). Previous studies have shown that DNFB or FITC applications can also be used to investigate ICD [20,26]. Our data suggests that a single exposure to both TNCB and DNFB can induce local inflammation, although the previously mentioned differences in histological image and immune cell populations suggest major differences. The thickened dermis, neutrophil accumulation, and lymphatic vessel dilation detected after a single 0.5% DNFB exposure suggest acute inflammation mediated by innate immunity, resembling the processes of ICD (Figure 1A–E, Supplementary Figure S2A–C). Notably, these processes might also correspond to the type IVd hypersensitivity reaction, with the hapten binding to preactivated T cell receptors and initiating inflammatory responses as described by the pharmacological interaction theory, although finding the exact underlying mechanism requires further research [10,27]. In addition, the severity of edema formation correlated with increased neutrophil accumulation; however, the underlying mechanism, as well as describing the severity of other symptoms of CD in relation to the applied hapten—including erythema or pruritus—requires additional studies. While it was previously reported that this initial inflammatory response in DNFB-induced mouse models is dose dependent and that its severity predetermines the seriousness of the reaction after repeated exposure, these conclusions mostly relied on ear thickness measurements [20]. We found that the repeated application of DNFB did not induce more-pronounced ear swelling than the first exposure; however, we only assessed the acute reaction, 24 h post-induction (Figure 3A–C). Of note, Weber and colleagues demonstrated that neutrophils participate in the sensitization phase through various processes [23]. Our findings regarding increased neutrophil infiltration after the first DNFB exposure, which did not induce aggravated inflammatory response after repeated exposure, highlight that these interactions might be hapten-dependent (Figure 1D,E and Figure 3D). Nonetheless, repeated DNFB application did significantly increase infiltrating regulatory T cell numbers compared with the first exposure, suggesting that the primary exposure induced peripheral tolerance-related processes (Figure 3D).

In contrast, a single exposure to TNCB elicited only mild, though still significant, ear swelling. However, professional antigen-presenting cells, including dendritic cells and macrophages, were more numerous after TNCB exposure than following DNFB, suggesting that this hapten is more prone to inducing classical immunization (Figure 1E). Additionally, repeated exposure to TNCB induced more-pronounced swelling and infiltration of neutrophils, helper, and cytotoxic T cells, more characteristic of the elicitation phase of a delayed-type hypersensitivity reaction (Figure 3A–D and Figure 4A,B) [26]. Our findings suggest that TNCB is more likely to induce Th1-mediated reactions resembling type IVa and IVc hypersensitivity; however, the involved immune pathways need additional studies [10]. These differences suggest that—at least in certain circumstances—DNFB acts more as an irritant, which could, at least partially, explain the differences in the effect of the ablation of LCs in the severity of CHS found with DNFB compared with TNCB and oxazolone [16,17,18].

Upon inspecting the lymph nodes, no major differences were found between the two haptens (Figure 2A–C). However, these experiments showed that the number of CD19+ B cells increased after the exposure to both allergens, possibly due to clonal expansion (Figure 2D). This finding is in line with results that demonstrated that contact allergens can act as adjuvants [28]. This result also supports the notion that B cells participate in the CHS reaction [29,30]. Additionally, it was demonstrated that the CD19 expression of B cells play an important anti-inflammatory role in CHS, and CD19-knockout mice present prolonged and more-severe symptoms [31]. Our findings show that CD19+ B cell recruitment is more pronounced after a single DNFB application than that after a single TNCB application, although no significant difference was found after repeated exposure. This difference might contribute to the finding that repeated exposure to DNFB did not increase the inflammatory response to the level observed with repeated TNCB application (Figure 1E and Figure 3D). Importantly, it was described that this immunomodulatory effect is mediated by a CD5+ subset of B cells; therefore, future research is recommended to describe these mechanisms with different frequently used model haptens [32].

Prop and colleagues reported decades ago that rats sensitized with DNFB and then challenged with TNCB presented more prominent ear swelling than naïve animals challenged with TNCB. In contrast, in our experiments with C57BL/6 mice, we found no significant differences in ear swelling or in dermal and epidermal thickness under similar regimens (Figure 3A–C) [21]. However, we observed that the TNCB challenge in DNFB-sensitized animals induced a significant increase in the number of infiltrating cytotoxic T cells compared with naïve animals exposed to the hapten. In addition, this regimen also increased total CD3+ T cell and CD4+ helper T cell recruitment (Figure 3D). Even though we could not confirm the cross-reaction based on ear thickness measurements, the increased accumulation of T cells suggests that DNFB-haptenized and TNCB-haptenized proteins may share common epitopes (Figure 3A–D) or have similar pharmacological effects on preactivated T cells [27].

In our recent study, we described that lymphatic vessels participate in the resolution of inflammation after the antigen challenge in the CHS model and found that repeated TNCB exposure can induce lymphatic vessel proliferation [22]. However, our current results showed that, while TNCB re-exposure resulted in an increased number of lymphatic vessels, the repeated application of DNFB did not induce this phenomenon (Figure 4A,B). This difference further emphasizes that these haptens elicit discrepant inflammatory processes upon repeated application. However, describing the molecular and cellular mechanisms responsible for this difference requires further research.

## 5. Conclusions

While the CHS mechanism is now well characterized, the allergen-specific immune responses remain only partially understood. Here, we demonstrated that both DNFB and TNCB induce inflammation upon first exposure; however, classical delayed-type hypersensitivity was more prominent with TNCB re-exposure. The underlying mechanisms responsible for these differences remain to be clarified and warrant further investigation. Elucidating these processes is essential for accurately interpreting experimental data and drawing clinically relevant, translatable conclusions. This information is crucial for advancing our understanding of the underlying pathomechanism of inflammatory skin diseases and for the development of innovative therapeutic strategies. Our results can contribute to the selection of the optimal model for researching distinct processes both in the sensitization and effector phases.

## Data Availability

Data is contained within the article or Supplementary Materials.

## Supplementary Materials

The following supporting information can be downloaded at https://www.mdpi.com/article/10.3390/biom15111540/s1: Figure S1: Gating strategy for flow cytometry analysis; Figure S2: Histological and cellular differences of the ear skin after a single TNCB or DNFB exposure; Figure S3: Visualization of infiltrating immune cells in the ear skin 24 h after hapten challenge.

## Abbreviations

The following abbreviations are used in this manuscript:

ACD	Allergic contact dermatitis
CD	Contact dermatitis
CHS	Contact hypersensitivity
DNFB	1-Fluoro-2,4-dinitrobenzene
FITC	Fluorescein isothiocyanate
ICD	Irritant contact dermatitis
LCs	Langerhans cells
TNCB	2-Chloro-1,3,5-trinitrobenzene
TNFR1	Tumor necrosis factor receptor 1

## References

[1] Li Y., Li L. (2021). Contact Dermatitis: Classifications and Management. Clin. Rev. Allergy Immunol..

[2] Nosbaum A., Vocanson M., Rozieres A., Hennino A., Nicolas J.F. (2009). Allergic and irritant contact dermatitis. Eur. J. Dermatol..

[3] Slodownik D., Lee A., Nixon R. (2008). Irritant contact dermatitis: A review. Australas. J. Dermatol..

[4] Hertl M., Bohlen H., Jugert F., Boecker C., Knaup R., Merk H.F. (1993). Predominance of epidermal CD8+ T lymphocytes in bullous cutaneous reactions caused by beta-lactam antibiotics. J. Investig. Dermatol..

[5] Martin S.F. (2004). T lymphocyte-mediated immune responses to chemical haptens and metal ions: Implications for allergic and autoimmune disease. Int. Arch. Allergy Immunol..

[6] Lepoittevin J.P. (2006). Metabolism versus chemical transformation or pro- versus prehaptens?. Contact Dermatitis.

[7] Christensen A.D., Haase C. (2012). Immunological mechanisms of contact hypersensitivity in mice. APMIS.

[8] Romani N., Clausen B.E., Stoitzner P. (2010). Langerhans cells and more: Langerin-expressing dendritic cell subsets in the skin. Immunol. Rev..

[9] Honda T., Egawa G., Grabbe S., Kabashima K. (2013). Update of immune events in the murine contact hypersensitivity model: Toward the understanding of allergic contact dermatitis. J. Investig. Dermatol..

[10] Posadas S.J., Pichler W.J. (2007). Delayed drug hypersensitivity reactions—New concepts. Clin. Exp. Allergy.

[11] Pichler W.J. (2019). Immune pathomechanism and classification of drug hypersensitivity. Allergy.

[12] Nakamura K., Aizawa M. (1981). Studies on the genetic control of picryl chloride contact hypersensitivity reaction in inbred rats. Transplant. Proc..

[13] Asherson G.L., Ptak W. (1968). Contact and delayed hypersensitivity in the mouse. I. Active sensitization and passive transfer. Immunology.

[14] Peszkowski M.J., Warfvinge G., Larsson A. (1994). Allergic and irritant contact responses to DNFB in BN and LEW rat strains with different TH1/TH2 profiles. Acta Derm. Venereol..

[15] Maibach H.I., Maguire H.C. (1963). Elicitation of delayed hypersensitivity (dncb contact dermatitis) in markedly panleukopenic guinea pigs. J. Investig. Dermatol..

[16] Kissenpfennig A., Henri S., Dubois B., Laplace-Builhé C., Perrin P., Romani N., Tripp C.H., Douillard P., Leserman L., Kaiserlian D. (2005). Dynamics and function of Langerhans cells in vivo: Dermal dendritic cells colonize lymph node areas distinct from slower migrating Langerhans cells. Immunity.

[17] Bennett C.L., van Rijn E., Jung S., Inaba K., Steinman R.M., Kapsenberg M.L., Clausen B.E. (2005). Inducible ablation of mouse Langerhans cells diminishes but fails to abrogate contact hypersensitivity. J. Cell Biol..

[18] Bennett C.L., Noordegraaf M., Martina C.A., Clausen B.E. (2007). Langerhans cells are required for efficient presentation of topically applied hapten to T cells. J. Immunol..

[19] Kneilling M., Mailhammer R., Hultner L., Schonberger T., Fuchs K., Schaller M., Bukala D., Massberg S., Sander C.A., Braumuller H. (2009). Direct crosstalk between mast cell-TNF and TNFR1-expressing endothelia mediates local tissue inflammation. Blood.

[20] Bonneville M., Chavagnac C., Vocanson M., Rozieres A., Benetiere J., Pernet I., Denis A., Nicolas J.F., Hennino A. (2007). Skin contact irritation conditions the development and severity of allergic contact dermatitis. J. Investig. Dermatol..

[21] Prop J., Griffiths A., Hutchinson I.V., Morris P.J. (1986). Specific suppressor T cells in rats active in the afferent phase of contact hypersensitivity. Cell. Immunol..

[22] Aradi P., Kovács G., Kemecsei É., Molnár K., Sági S.M., Horváth Z., Mehrara B.J., Kataru R.P., Jakus Z. (2024). Lymphatic-Dependent Modulation of the Sensitization and Elicitation Phases of Contact Hypersensitivity. J. Investig. Dermatol..

[23] Weber F.C., Nemeth T., Csepregi J.Z., Dudeck A., Roers A., Ozsvari B., Oswald E., Puskas L.G., Jakob T., Mocsai A. (2015). Neutrophils are required for both the sensitization and elicitation phase of contact hypersensitivity. J. Exp. Med..

[24] Szotak-Ajtay K., Szoke D., Kovacs G., Andreka J., Brenner G.B., Giricz Z., Penninger J., Kahn M.L., Jakus Z. (2020). Reduced Prenatal Pulmonary Lymphatic Function Is Observed in Clp1 (K/K) Embryos With Impaired Motor Functions Including Fetal Breathing Movements in Preparation of the Developing Lung for Inflation at Birth. Front. Bioeng. Biotechnol..

[25] Szoke D., Kovacs G., Kemecsei E., Balint L., Szotak-Ajtay K., Aradi P., Styevkone Dinnyes A., Mui B.L., Tam Y.K., Madden T.D. (2021). Nucleoside-modified VEGFC mRNA induces organ-specific lymphatic growth and reverses experimental lymphedema. Nat. Commun..

[26] Saint-Mezard P., Krasteva M., Chavagnac C., Bosset S., Akiba H., Kehren J., Kanitakis J., Kaiserlian D., Nicolas J.F., Berard F. (2003). Afferent and efferent phases of allergic contact dermatitis (ACD) can be induced after a single skin contact with haptens: Evidence using a mouse model of primary ACD. J. Investig. Dermatol..

[27] Pichler W.J. (2002). Pharmacological interaction of drugs with antigen-specific immune receptors: The p-i concept. Curr. Opin. Allergy Clin. Immunol..

[28] Le Borgne M., Etchart N., Goubier A., Lira S.A., Sirard J.C., van Rooijen N., Caux C., Ait-Yahia S., Vicari A., Kaiserlian D. (2006). Dendritic cells rapidly recruited into epithelial tissues via CCR6/CCL20 are responsible for CD8+ T cell crosspriming in vivo. Immunity.

[29] Tsuji R.F., Szczepanik M., Kawikova I., Paliwal V., Campos R.A., Itakura A., Akahira-Azuma M., Baumgarth N., Herzenberg L.A., Askenase P.W. (2002). B cell-dependent T cell responses: IgM antibodies are required to elicit contact sensitivity. J. Exp. Med..

[30] Askenase P.W., Kawikova I., Paliwal V., Akahira-Azuma M., Gerard C., Hugli T., Tsuji R. (1999). A new paradigm of T cell allergy: Requirement for the B-1 cell subset. Int. Arch. Allergy Immunol..

[31] Watanabe R., Fujimoto M., Ishiura N., Kuwano Y., Nakashima H., Yazawa N., Okochi H., Sato S., Tedder T.F., Tamaki K. (2007). CD19 expression in B cells is important for suppression of contact hypersensitivity. Am. J. Pathol..

[32] Yanaba K., Bouaziz J.D., Haas K.M., Poe J.C., Fujimoto M., Tedder T.F. (2008). A regulatory B cell subset with a unique CD1dhiCD5+ phenotype controls T cell-dependent inflammatory responses. Immunity.

