# Supplementary material for: Hapten-Specific Cellular Immune Responses in the Elicitation and Sensitization Phases of Murine Contact Hypersensitivity
Source: Biomolecules. 2025 Nov 1;15(11):1540. doi: 10.3390/biom15111540 (PMC12650261; doi:10.3390/biom15111540)
Supplement: Supplementary file 1 [file biomolecules-15-01540-s001.zip › biomolecules-3895530-supplementary.pdf]

SUPPLEMENTARY MATERIAL

Hapten-Specific Cellular Immune Responses in the Elicitation and Sensitization Phases of Murine Contact Hypersensitivity

Kornél Molnár †, Gábor Kovács †, Bence Kormos, Petra Aradi and Zoltán Jakus *

Department of Physiology, Semmelweis University, Budapest H-1094, Hungary;

molnar.kornel@semmelweis.hu (K.M.); kovacs.gabor@semmelweis.hu (G.K.);

kormos.bence04@stud.semmelweis.hu (B.K.); aradi.petra@semmelweis.hu (P.A.)

* Correspondence: jakus.zoltan@semmelweis.hu

† These authors contributed equally to this work.

Keywords: contact hypersensitivity; mouse models; inflammation; haptens; TNCB; DNFB; dermatitis

Correspondence

Zoltán Jakus MD, PhD

Associate Professor

Department of Physiology

Semmelweis University

Tuzolto utca 37-47

1094 Budapest, Hungary

Tel:(+36-1)-459-1500 x60-429

E-mail: jakus.zoltan@semmelweis.hu

SUPPLEMENTARY FIGURES AND LEGENDS

Gating strategy

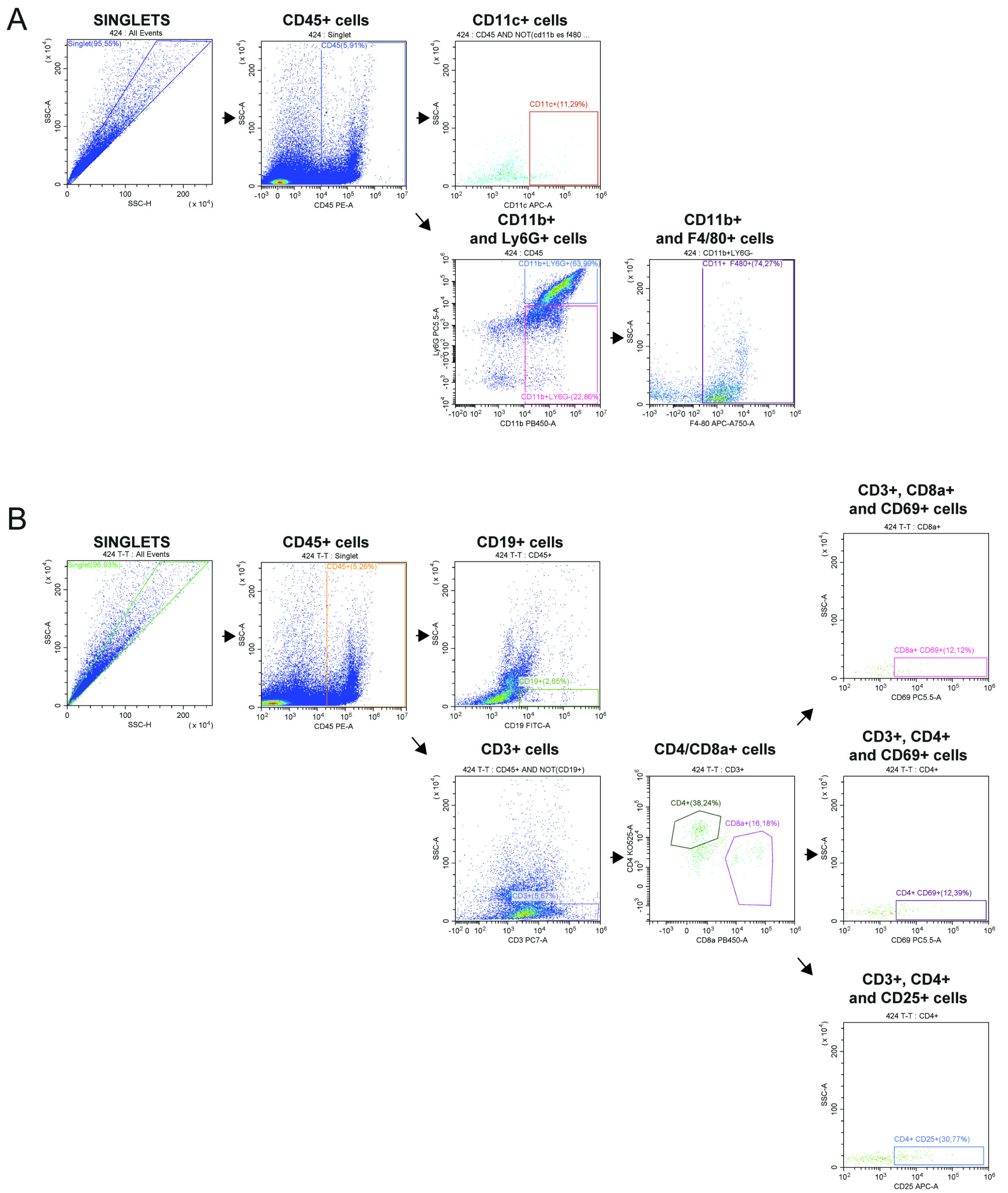

Supplementary Figure S1. Gating strategy for flow cytometry analysis. The gating strategy for the analysis of cells of **(A)** innate immunity and **(B)** adaptive immunity. Dot plots represent the applied gates and arrows indicate the parent populations from which the subsequent gating was performed.

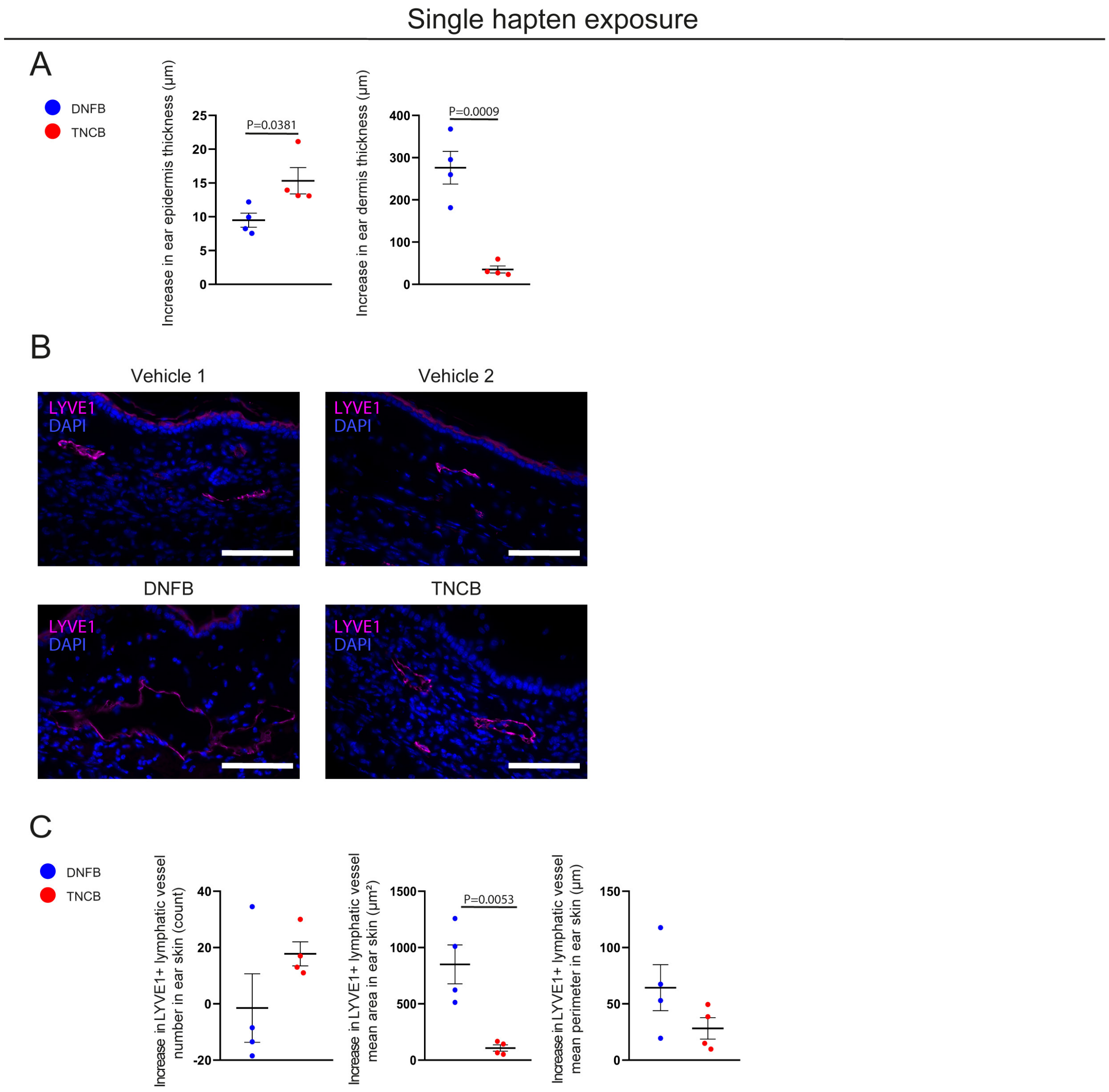

Supplementary Figure S2. Histological and cellular differences of the ear skin after a single TNCB or DNFB exposure. **(A)** Quantification of the dermis and epidermis layers from H&E-stained sections of ears collected after a single hapten exposure (Unpaired t-test; mean ± SEM; n= 4 for each group). **(B)** Anti-LYVE-1 immunofluorescence staining of paraffin-based sections of ears 24 hours after single exposure (Bars= 100 µm; n= 4 for each group). **(C)** Quantification of lymphatic vessel mean perimeter, mean area and number from anti-LYVE-1 immunofluorescence images of ears collected 24 hours after a single hapten exposure (Unpaired t-test; mean ± SEM; n= 4 for each group).

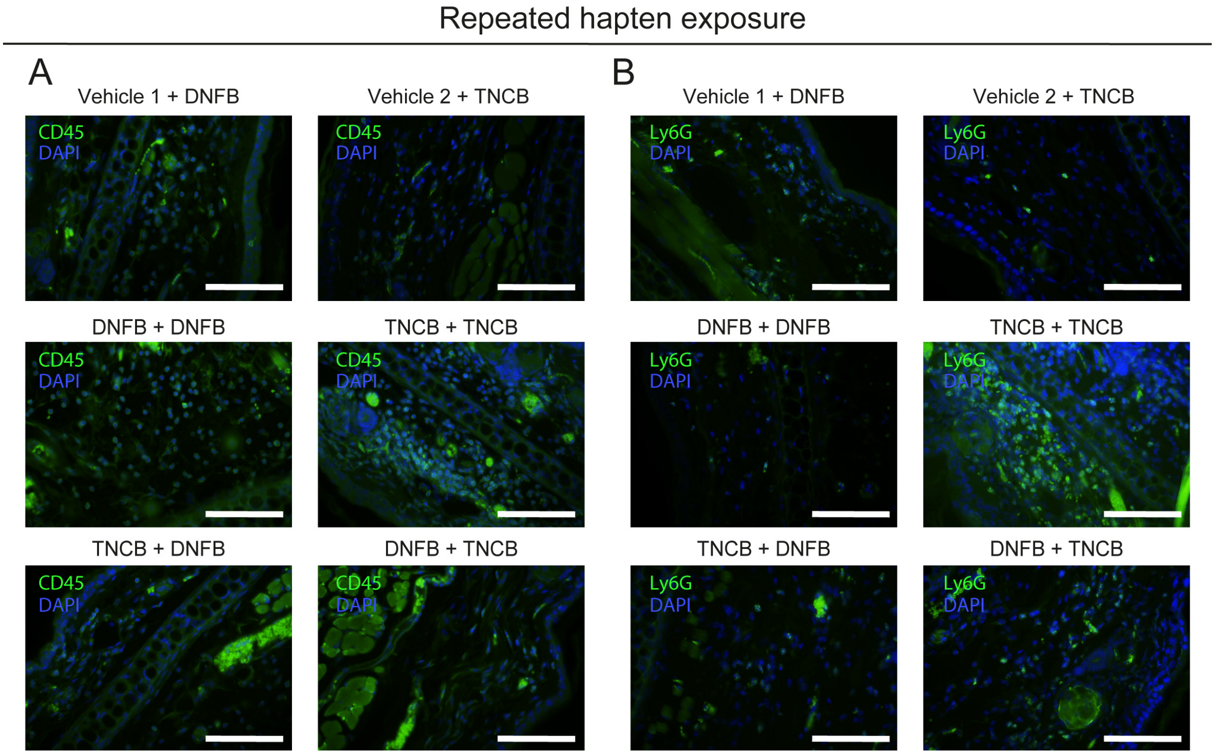

Supplementary Figure S3. Visualization of infiltrating immune cells in the ear skin 24 hours after hapten challenge. **(A)** Anti-CD45 immunofluorescence staining of paraffin-based sections of ears 24 hours after hapten challenge (Bars= 100 µm; n= 6-8 for each group). **(B)** Anti-Ly6G immunofluorescence staining of paraffin-based sections of ears 24 hours after hapten challenge (Bars= 100 µm; n= 7-8 for each group)
